# Preprocessing Unevenly Sampled RR Interval Signals to Enhance Estimation of Heart Rate Deceleration and Acceleration Capacities in Discriminating Chronic Heart Failure Patients from Healthy Controls

**DOI:** 10.1155/2020/9763826

**Published:** 2020-03-31

**Authors:** Ping Cao, Bailu Ye, Linghui Yang, Fei Lu, Luping Fang, Guolong Cai, Qun Su, Gangmin Ning, Qing Pan

**Affiliations:** ^1^College of Information Engineering, Zhejiang University of Technology, 288 Liuhe Road, Hangzhou 310023, China; ^2^Zhijiang College, Zhejiang University of Technology, Shaoxing 312030, China; ^3^Department of ICU, Zhejiang Hospital, 12 Lingyin Road, Hangzhou 310013, China; ^4^Department of ICU, First Affiliated Hospital Zhejiang University, 79 Qingchun Road, Hangzhou 310003, China; ^5^Department of Biomedical Engineering, Key Laboratory of Biomedical Engineering of Ministry of Education, Zhejiang University, 38 Zheda Road, Hangzhou 310027, China

## Abstract

*Objective*. The deceleration capacity (DC) and acceleration capacity (AC) of heart rate, which are recently proposed variants to the heart rate variability, are calculated from unevenly sampled RR interval signals using phase-rectified signal averaging. Although uneven sampling of these signals compromises heart rate variability analyses, its effect on DC and AC analyses remains to be addressed. *Approach*. We assess preprocessing (i.e., interpolation and resampling) of RR interval signals on the diagnostic effect of DC and AC from simulation and clinical data. The simulation analysis synthesizes unevenly sampled RR interval signals with known frequency components to evaluate the preprocessing performance for frequency extraction. The clinical analysis compares the conventional DC and AC calculation with the calculation using preprocessed RR interval signals on 24-hour data acquired from normal subjects and chronic heart failure patients. *Main Results*. The assessment of frequency components in the RR intervals using wavelet analysis becomes more robust with preprocessing. Moreover, preprocessing improves the diagnostic ability based on DC and AC for chronic heart failure patients, with area under the receiver operating characteristic curve increasing from 0.920 to 0.942 for DC and from 0.818 to 0.923 for AC. *Significance*. Both the simulation and clinical analyses demonstrate that interpolation and resampling of unevenly sampled RR interval signals improve the performance of DC and AC, enabling the discrimination of CHF patients from healthy controls.

## 1. Introduction

Assessing the autonomic nervous system (ANS) activity is crucial for analyses such as risk prediction of mortality after myocardial infarction [1], diagnosis of chronic heart failure (CHF) [2, 3], and prediction of diabetic neuropathy [4, 5]. Although the heart rate variability (HRV) has been widely used to assess the ANS activity in the last decades [6–8], its ability to distinguish activity from the sympathetic and parasympathetic nervous systems is limited [9]. In fact, most time-domain indices, such as standard deviation of all normal-to-normal intervals, measure the overall ANS activity, and the relation of frequency-domain indices with separate limbs in the ANS remains controversial [6]. New indices obtained from RR interval (RRI) signals have been developed for improved characterization of the ANS activity. For instance, the deceleration capacity (DC) and acceleration capacity (AC) of the heart rate, which were proposed a decade ago, are promising for assessing the ANS activity [10]. They aim to separately characterize deceleration and acceleration components in HRV, outperforming conventional HRV indices in prediction of mortality and risk stratification of postmyocardial infarction patients [11–13].

The DC and AC can be computed from an RRI signal using phase-rectified signal averaging (PRSA) [14], which extracts quasiperiodicities from nonstationary sequential signals. Specifically, given a sequential signal, PRSA first selects decelerating and accelerating points as anchors. Then, segments surrounding the anchors are defined, aligned, and averaged to obtain a PRSA curve. Finally, wavelet analysis is applied to quantify the characteristic oscillations in the PRSA curve. When the algorithm is applied to RRI signals, DC and AC correspond to the wavelet coefficients from the center of the PRSA curve. PRSA aims to eliminate noise influence by averaging phase-synchronized segments, and the wavelet transform quantifies the averaged signal. Hence, the quality of phase synchronization is a determinant factor in PRSA.

Several PRSA variants have been proposed to enhance the estimation accuracy of DC and AC [15–18]. Specifically, we modified PRSA using a stricter criterion of anchor point selection to enhance phase synchronization [15]. Likewise, Arsenos and Manis [16] modified anchor point selection and its application in PRSA to avoid nonphysiological negative DC values. Other PRSA modifications measure transient velocity changes or exclude nonvagally mediated rhythms [17, 18]. Still, most studies for refining the DC and AC calculation focus on rectifying phase synchronization, but the effect of preprocessing RRI signals, which is essential in the estimation of conventional HRV indices [19], has been mostly neglected.

The DC and AC are usually calculated from raw unevenly sampled RRI signals, following the original proposal [20]. However, unevenly spaced samples may compromise HRV analysis in the frequency domain [19]. Therefore, we aimed to evaluate the effect of preprocessing RRI signals on diagnosis based on DC and AC through both simulation and clinical analyses. The simulation analysis synthesizes unevenly sampled RRI signals with known frequency components, and a subsequent wavelet analysis illustrates the effect of preprocessing on the extraction of characteristic frequency components. In the clinical analysis, the preprocessing is applied to real RRI datasets for distinguishing CHF patients from the healthy controls.

## 2. Materials and Methods

### 2.1. Datasets

#### 2.1.1. Synthetic Dataset

We generated synthetic RRI signals with physiologically feasible frequency components, which mainly comprise a low-frequency range of 0.04–0.15 Hz and a high-frequency range of 0.15–0.4 Hz [9], with central frequencies of approximately 0.095 Hz and 0.275 Hz, respectively. Therefore, the synthetic continuous RRI signal was generated as follows:

(1) $$\begin{array}{c} \text{RRI}\left(t\right)={\text{RRI}}_{\text{mean}}+A_{1}\sin\left(2\pi f_{1}t\right)+A_{2}\sin\left(2\pi f_{2}t\right), \end{array}$$

where RRI_mean_ represents the average RRI, *f*_1_=0.095 Hz, *f*_2_=0.275 Hz, and *A*_1_ and *A*_2_ are the amplitudes of the two respective frequency components. We examined three levels of RRI_mean_, namely, 1000 ms, 667 ms, and 500 ms, corresponding to heart rates of 60 bpm, 90 bpm, and 120 bpm. For each RRI_mean_ level, we generated 200 RRI signals of 2 hours per signal. The basal values of *A*_1_ and *A*_2_ were set to 55 ms and 44 ms, respectively. These values were randomized within ±10% of their basal values for simulation. To resemble the nonstationarity of the RRI, we added phase jumps *φ*(*φ*=2*πr*_1_) every 4 periods and frequency jumps Δ*f*(Δ*f*=0.05*fr*_2_) every 20 periods according to the method in [14], where *r*_1_ and *r*_2_ are random numbers following a normal distribution. Therefore, equation (1) becomes

(2) $$\begin{array}{c} \text{RRI}\left(t\right)={\text{RRI}}_{\text{mean}}+A_{1}\sin\left[2\pi \left(f_{1}+\Delta f_{1}\right)t+\varphi_{1}\right]+A_{2}\sin\left[2\pi {\left(f_{2}+\Delta f\right)}_{2}t+\varphi_{2}\right]. \end{array}$$

We then prepared an unevenly sampled signal RRI_*n*_ from each generated continuous series RRI(*t*) following the approach proposed by Clifford [21]. We recorded the first time RRI pair (*t*_1_, RRI(1)) as point pair (*t*_1_′, RRI_1_) of the unevenly sampled signal. Then, we evaluated each sample pair (*t*_*i*_, RRI(*i*)) in the continuous RRI signal. If relationship

(3) $$\begin{array}{c} t_{i}-t_{n-1}^{\prime }\geq \text{RRI}\left(i\right) \end{array}$$

was satisfied, we recorded point pair *n* as (*t*_*n*_′=*t*_*i*_, RRI_*n*_=RRI(*i*)). We denote a synthetic RRI signal as RRI_s_.

The power spectral density was obtained using the Welch method [21] to determine the validity of each synthetic raw RRI signal considering a 5-minute segment, which was interpolated and resampled. We obtained a low-to-high frequency ratio of 1.70 ± 0.29, being consistent with the physiological range of 1.5–2.0 [9]. The absolute power of LF and HF components also lies in the physiologically valid range [9].

#### 2.1.2. Clinical Dataset

RRI recordings of the healthy subjects and CHF patients over 24 h were obtained from PhysioNet (http://www.physionet.org). Data from healthy subjects were retrieved from the Normal Sinus Rhythm RR Interval Database and the MIT-BIH Normal Sinus Rhythm Database. Data from the CHF patients were retrieved from two databases, the Congestive Heart Failure RR Interval Database and the BIDMC Congestive Heart Failure Database. Two cases in the CHF dataset and one case in the healthy subject dataset were discarded due to the presence of continuous premature atrioventricular contractions. The sampling frequency for the electrocardiogram signals was 250 Hz for the BIDMC Congestive Heart Failure Database and 128 Hz for the other three datasets. Further details of the datasets are listed in Table 1.

### 2.2. DC and AC Calculation

#### 2.2.1. PRSA Analysis

PRSA can extract quasiperiodicities from nonstationary signals [14, 20], and hence the DC and AC can be calculated by applying PRSA to RRI signals. The PRSA algorithm for DC proceeds as follows. First, all sample points from an RRI signal are checked, and its *i-*th point is selected as anchor if it satisfies

(4) $$\begin{array}{c} {\text{RRI}}_{i}>{\text{RRI}}_{i-1}. \end{array}$$

Then, a segment is defined around each anchor point with length 2*L* + 1, where *L* is the length of the series at each side of the anchor. Segment *S*_*i*_ is defined as

(5) $$\begin{array}{c} S_{i}=\left\{{\text{RRI}}_{i-L},{\text{RRI}}_{i-L+1},\ldots ,{\text{RRI}}_{i},\ldots ,{\text{RRI}}_{i+L-1},{\text{RRI}}_{i+L}\right\}, \end{array}$$

and *L* should allow the inclusion of the slowest oscillation from the interest signal [14]. In general, *L* is set to 60 for raw RRI signals according to [10]. Finally, all the segments are aligned by the anchor points, and the PRSA curve is obtained as the average across segments as

(6) $$\begin{array}{c} \bar{\text{RRI}}\left(p\right)=\frac{1}{N}\sum_{k=1}^{N}{\text{RRI}}_{i_{k}+p},\;\;p=-L,-L+1,\ldots ,0,\ldots ,L-1,L\text{,} \end{array}$$

where $\bar{\text{RRI}}\left(p\right)$ is the averaged RRI signal obtained after PRSA, *N* is the number of segments, *p* indicates the signal index, and *i*_*k*_ is the anchor point of the *k*-th segment. The AC calculation only differs in the condition to select the anchor points:

(7) $$\begin{array}{c} {\text{RRI}}_{i}<{\text{RRI}}_{i-1}. \end{array}$$

As the segments are phase synchronized based on equations (4) and (7), averaging eliminates noise while maintaining quasiperiodicities.

#### 2.2.2. Quantification of PRSA Curve

Wavelet analysis with Gaussian basis is widely used for the quantitative characterization of the PRSA curve [14, 20]. However, when PRSA is applied to obtain the DC and AC clinical indices, the Haar wavelet is used instead of the Gaussian wavelet to simplify and speed up the calculation, according to the original proposal of the indices [20]. Therefore, we adopted a third-order Gaussian wavelet and the Haar wavelet for quantifying the PRSA curves at different scales and positions in simulation and the Haar wavelet for calculations on clinical data.

To quantitatively characterize the PRSA curve, the squared wavelet coefficients were computed to determine the degree of oscillation at different scales and positions. At a specific position, pseudofrequency *f*_*p*_ that a wavelet represents at scale *s* can be computed as

(8) $$\begin{array}{c} f_{p}\approx \frac{F_{c}}{s\cdot \Delta t}, \end{array}$$

where *F*_*c*_ is the central frequency of the wavelet basis function and Δ*t* is the sampling period of the signal. If wavelet analysis is directly applied to unevenly sampled RRI signals, Δ*t* is replaced by the average RRI (RRI_mean_) as approximation. Then, equation (8) becomes

(9) $$\begin{array}{c} f_{p}\approx \frac{F_{c}}{s\cdot {\text{RRI}}_{\text{mean}}}. \end{array}$$

The Haar wavelet was selected to calculate the DC and AC in clinical data as follows [10, 20]:

(10) $$\begin{array}{c} \text{DC}\left(\text{AC}\right)=\sum_{p=-L}^{L}\bar{\text{RRI}}\left(p\right)\frac{h\left(p/s\right)}{s}, \end{array}$$

where *h*(*t*) represents the Haar wavelet given by

(11) $$\begin{array}{c} h\left(t\right)=\left\{\begin{array}{cc} -1/2, & -1\leq t<0,\\ +1/2, & \;0\leq t<1,\\ 0, & \text{otherwise}. \end{array}\right. \end{array}$$

For conventional DC and AC calculation, the scale is set to 2 according to a large-scale clinical trial for predicting mortality of postmyocardial infarction patients [10]. In this paper, the conventional DC and AC (scale of 2 and computed from raw RRI signals) are denoted with subscript “conv,” and equation (10) can be rewritten as

(12) $$\begin{array}{c} {\text{DC}}_{\text{conv}}\left({\text{AC}}_{\text{conv}}\right)=\frac{\left[\bar{\text{RRI}}\left(0\right)+\bar{\text{RRI}}\left(1\right)-\bar{\text{RRI}}\left(-1\right)-\bar{\text{RRI}}\left(-2\right)\right]}{4}. \end{array}$$

### 2.3. Preprocessing: Interpolation and Resampling

Interpolation and resampling are common preprocessing steps for unevenly sampled RRI signals before time- and frequency-domain HRV computation [19]. In this study, each RRI signal was linearly interpolated to form a continuous signal. Then, the interpolated signal was resampled to obtain an evenly sampled RRI signal. Preprocessing was applied to both the synthetic unevenly sampled RRI signals and clinical data. For the synthetic and clinical data, the resampling frequencies were 4 Hz and 2–7 Hz (with increments of 1 Hz), respectively.

As resampling changes the period between consecutive RRIs, according to equation (8), we should determine a new scale according to the resampling frequency to guarantee that wavelet coefficients reflect the target frequency components. Therefore, we scanned the scale from 1 to 100 for the simulation analysis and the clinical analysis, respectively. A preprocessed RRI signal is denoted as RRI_p_.

For the preprocessed RRI signal, *L* was set according to resampling frequency *f*_r_. Considering the heart rate range from 60 bpm to 120 bpm, *L* was set to 60 × *f*_r_ to guarantee that the PRSA curve from a preprocessed RRI signal reflects the slowest fluctuations.

### 2.4. Data Analysis

We compared the conventional DC and AC calculation with the calculation from the preprocessed RRI signals and between healthy and CHF subjects using either the Student's *t*-test for normally distributed data or the Wilcoxon signed-rank test for other distributions. The receiver operating characteristic allowed to evaluate the accuracy of the indices per combination of resampling frequency and wavelet scale, and the area under the receiver operating characteristic curve (AUC) was obtained. The modifications of DC and AC calculation [15–17] described in Table 2 were also evaluated on raw and preprocessed RRI signals. The AUC, sensitivity, specificity, and accuracy under certain cutoff values were compared. The data analysis was implemented on SigmaPlot 10.0 (Systat Software, San Jose, CA, USA) at statistical significance level *p* < 0.001.

## 3. Results

### 3.1. Synthetic Data

**Figure 1:**
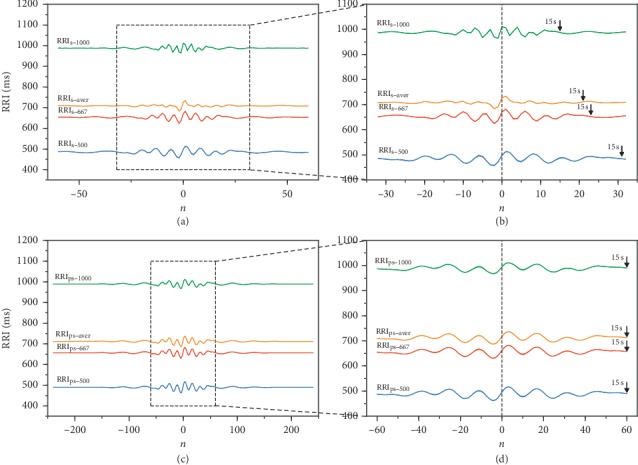
PRSA curves of synthetic RRI signals at three levels of RRI and average of all RRI signals. (a) PRSA curves of synthetic raw RRI signals. (b) Enlarged view of central oscillations in (a) with window of 15 s for RRI_s-500_. (c) PRSA curves of preprocessed synthetic RRI signals at resampling frequency of 4 Hz. (d) Enlarged view of central oscillations in (c) with window of 15 s for the three curves.

The PRSA curves of the raw and preprocessed synthetic RRI signals at three RRI levels and their average level are shown in Figure 1. When using raw RRI signals, the oscillation lengths at the same frequency vary according to the heart rate, with slower rates presenting shorter oscillations. In contrast, the lengths are equal when using the preprocessed RRI signals. For the average PRSA curve, the central oscillations are slightly attenuated because the segments are synchronized at the center. In contrast, surrounding oscillations are largely damped probably by the varying period between consecutive RRIs according to the average value, leading to phase asynchrony among segments and elimination of quasiperiodicities. On the other hand, the oscillations were maintained in the average PRSA curves obtained from preprocessed RRI signals because the curves with different average RRIs have the same period between consecutive RRIs.

**Figure 2:**
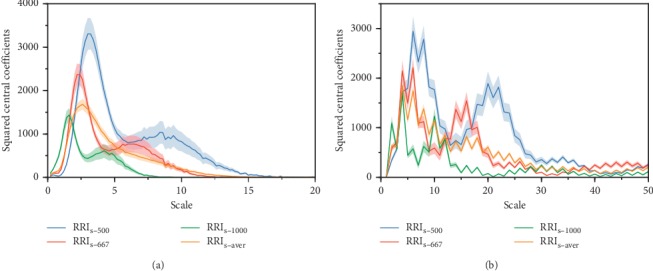
Squared central wavelet coefficients of PRSA curves at different scales for conventional DC and AC calculation. The shaded area represents the variation range of each curve. (a) Wavelet analysis using the third-order Gaussian wavelet. (b) Wavelet analysis using the Haar wavelet.

**Figure 3:**
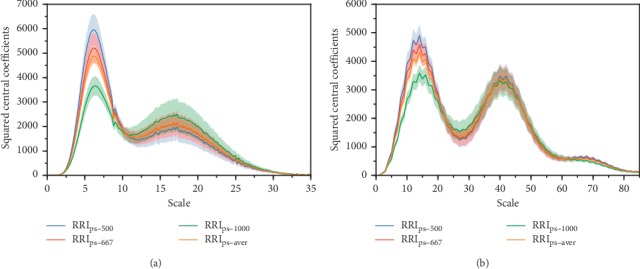
Squared central wavelet coefficients of PRSA curves obtained after interpolation and resampling at different scales. The shaded area represents the variation range of each curve. (a) Wavelet analysis using the third-order Gaussian wavelet. The maximum coefficients occur at scale *s* = 6.2. (b) Wavelet analysis using the Haar wavelet. The maximum coefficients occur at scale *s* = 14.0.

The wavelet analysis was applied to all the obtained groups of PRSA curves in Figure 1. The central squared wavelet coefficients at each scale are depicted in Figures 2 and 3 for the unevenly sampled and preprocessed synthetic signals, respectively. As DC and AC reflect high frequencies in the HRV [22], we focused on its corresponding peak (peak at smaller scale). In Figure 2, the peak position varies according to the average RRI. For RRI_mean_ of 500 ms, 667 ms, and 1000 ms, the peaks in Figure 2(a) are located at *s*_500_ = 3.0, *s*_667_ = 2.2, and *s*_1000_ = 1.6 with frequencies of 0.267 Hz, 0.273 Hz, and 0.250 Hz, respectively, and the peaks in Figure 2(b) are located at *s*_500_ = 6.0, *s*_667_ = 6.0, and *s*_1000_ = 4.0 with frequencies of 0.332 Hz, 0.249 Hz, and 0.249 Hz, respectively. In contrast, the peaks from the preprocessed signals are located at the same scale *s* = 6.2 (Figure 3(a)) and *s* = 14.0 (Figure 3(b)), resulting in pseudofrequencies of 0.258 Hz and 0.285 Hz according to equation (8). We consider that in both analyses, the pseudofrequency is 0.275 Hz in the synthetic signal, and the slight deviations are probably due to *F*_*c*_ in equation (8) being an approximated value, as a wavelet is not a purely sinusoidal signal. In addition, the frequency jumps added to the synthetic signals may lead to pseudofrequency deviations.

### 3.2. Clinical Data

**Figure 4:**
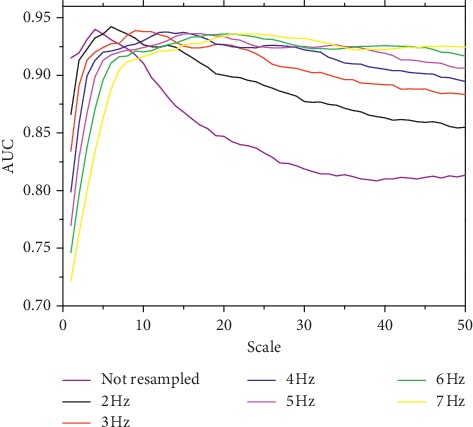
AUC of DC obtained from clinical data at different scales with and without preprocessing.

**Figure 5:**
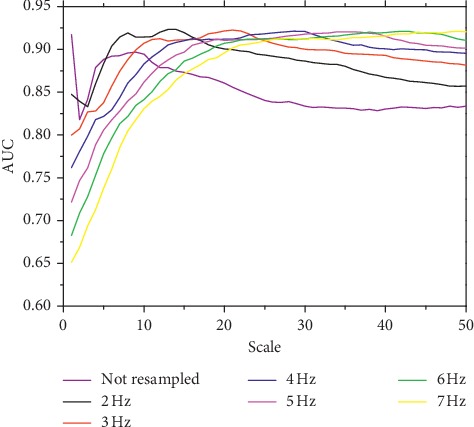
AUC of AC obtained from clinical data at different scales with and without preprocessing.

The effect of preprocessing was further examined on clinical data. Figures 4 and 5 show the AUC at different resampling frequencies and wavelet scales for DC_p_ and AC_p_, respectively. The optimal AUCs at different resampling frequencies are close, as listed in Table 3, with the global optimal resampling frequency being 2 Hz for both DC_p_ and AC_p_. The highest AUCs for DC_p_ and AC_p_ are 0.942 and 0.923 with optimal wavelet scales of 6 and 14, respectively. The AUCs for the DC and AC computed from raw RRI signals with optimized scale are close with the highest AUCs for DC_p_ and AC_p_, respectively. Table 3 also shows that the optimal scale increases with higher resampling frequency, at a relatively constant pseudofrequency for both DC_p_ and AC_p_. Still, DC_p_ was most sensitive at approximately 0.3 Hz, and the frequency that best reflects AC_p_ was approximately 0.14 Hz. We then compared DC_conv_ and AC_conv_ with the indices calculated from the preprocessed RRI signals using the optimal parameters. Their values for the healthy and CHF subjects are listed in Table 4.

**Figure 6:**
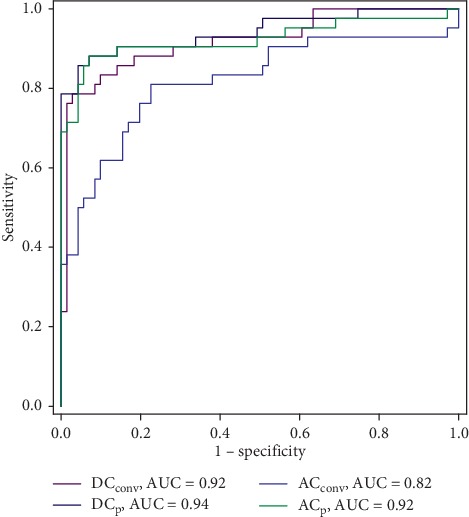
Receiver operating characteristic curves of DC and AC calculated from raw and preprocessed RRI signals.

The receiver operating characteristic curves of DC_conv_, DC_p_, AC_conv_, and AC_p_ are depicted in Figure 6. DC and AC calculated from preprocessed RRI signals outperform the conventional calculations. Appropriate cutoff values were selected according to the highest values from the sum of sensitivity and specificity. The sensitivity, specificity, and diagnostic accuracy for the indices including the existing calculation variants are listed in Table 5. The optimal parameters from the existing and improved calculation method after preprocessing correspond to those from DC_p_ or AC_p_.

## 4. Discussion

Previous studies have suggested to use unevenly sampled RRI signals for PRSA because interpolation and resampling may induce artifacts [20]. Therefore, raw RRI signals have been used for conventional DC and AC calculation. However, we found that wavelet analysis may not be appropriate for raw RRI signals because they are unevenly sampled, thus distorting the wavelet analysis. In this study, simulation and clinical analyses demonstrated that interpolation and resampling of unevenly sampled RRI signals improve the performance of DC and AC calculation.

The DC and AC, which are computed as wavelet coefficients at a fixed scale, extract specific frequency components from RRI signals. However, our simulation analysis demonstrates that the effectiveness of wavelet coefficients may be degraded regarding the extraction of target characteristic frequencies if the signals are unevenly sampled. For synthetic signals with different average RRIs (i.e., 500 ms, 667 ms, and 1000 ms), the third-order Gaussian wavelet analysis shows differing scales in Figure 2(a) (3.0, 2.2, and 1.6 with pseudofrequencies of 0.267 Hz, 0.273 Hz, and 0.250 Hz, respectively) corresponding to the known frequency of 0.275 Hz. Similar finding is observed when performing Haar wavelet analysis. Figure 2(b) shows scales of 6.0, 6.0, and 4.0 with pseudofrequencies of 0.332 Hz, 0.249 Hz, and 0.249 Hz, respectively. The unique scale obtained for RRI_s-500_ and RRI_s-667_ is probably due to the discontinuous nature of the Haar wavelet. Even so, we found similar shift of the whole curve from RRI_s-500_ to RRI_s-667_ as compared with the curves obtained by Gaussian wavelet. Therefore, when using a fixed scale, such as that used in the DC_conv_ and AC_conv_ calculation, the wavelet coefficients of the PRSA curves with different average RRIs determine the frequency power with deviation from the target frequency. In other words, the obtained indices may not reflect the full power of the target frequency components, consequently impairing the evaluation of ANS activity, as suggested by a previous power spectral analysis [23]. In contrast, after interpolation and resampling, the wavelet coefficients at fixed scale reflect the full power of the target frequencies regardless of the average RRI (Figure 3).

The simulation analysis also suggests that the DC and AC may reflect oscillations at biased frequency values if the RRI is not properly preprocessed. Hence, the ability of the indices to reflect specific high frequencies in the HRV may be compromised, as confirmed by the clinical analysis. For both DC and AC, the calculation using preprocessed RRI signals has improved accuracy. In fact, the conventional DC calculation retrieves an AUC of 0.920, whereas the calculation from preprocessed RRI signals yields a higher AUC of 0.942. Similarly, the AUC of the AC increases from 0.818 to 0.923. It is noteworthy that the AUCs of the optimized DC and AC computed from the raw RRI signals are 0.940 and 0.917, respectively, which are greater than the conventional DC and AC, and are only slightly worse than the indices computed from the preprocessed RRI signals. Even so, we suggest that the interpolation and resampling are necessary steps for deriving more robust indices as they render more stable performance of DC and AC with varying scales, as observed in Figures 4 and 5. It is particularly important when the scale optimization is based on a small dataset, in which the optimized scale may be deviated from the real optimal scale. Table 3 shows that the optimal scale increases at higher resampling frequency with both ratio and pseudofrequency remaining relatively constant. Hence, preprocessing is effective and insensitive to the resampling frequency at a suitable scale.

Existing variants of the DC and AC calculation focus on improving phase synchronization [15–17] but neglect the influence of unevenly sampled RRI signals. A comparison to these variants demonstrates the effectiveness of preprocessing. DC_conv_ and AC_conv_ calculated from preprocessed RRI signals (DC_p_ and AC_p_) outperform most variants obtained from unevenly sampled RRI signals, except for DC_p_ compared to DC_m1_ (Table 5). When we applied the variants to the preprocessed RRI signals, most AUC values improved (except for DC_m1_). Hence, the calculation of DC and AC from preprocessed RRI signals may improve the estimation performance and ability to distinguish CHF from healthy subjects.

Nevertheless, the insufficient number of clinical subjects limits the scope of this study. Large-scale datasets are required to confirm our findings. In addition, as no complementary information about the patients was available, we were not able to analyze the physiological background relation to the calculation improvement. Moreover, the low sampling frequency of electrocardiography may introduce noise in the RRI signals, possibly biasing the calculation results. Another limitation is the simplification of RRI signals for simulation. The adopted method facilitates the generation of signals with known frequencies but fails to introduce physiological regulations that affect the RRI. For example, a very low-frequency component reflecting long-term regulatory circuits, such as thermoregulation, renin-angiotensin system activity, and peripheral sympathetic vasomotor control, was not modeled [24]. Hence, more realistic RRI generation methods, such as the integral pulse frequency modulator [25] and the cardiovascular model [26, 27], which considers short-term sympathovagal regulation of the heart cycle, are required for improving our analyses. Finally, note that we cannot provide conclusive evidence on the possible improvement of the proposed preprocessing for improving risk stratification based on DC and AC from the clinical results because only healthy subjects and CHF patients were compared.

## 5. Conclusion

We investigated the effect of interpolating and resampling unevenly sampled RRI signals on the accuracy and diagnostic ability of DC and AC. The simulation analysis demonstrates that conventional DC and AC calculation retrieves different target frequencies depending on the average RRI, but a single target frequency is obtained after signal preprocessing. The clinical analysis suggests that interpolation and resampling improve the identification of CHF patients based on the DC and AC. Our findings suggest the effectiveness of preprocessing unevenly sampled RRI signals before DC and AC calculation to improve the diagnostic performance of CHF based on these indices.

## Figures and Tables

**Table 1:** Details of datasets from CHF patients and healthy subjects (control group).

	Database	No. of subjects	Age (years)	NYHA classification
CHF	Congestive Heart Failure RR Interval Database	29	55.0 ± 11.9	I–III
	BIDMC Congestive Heart Failure Database	15	56.0 ± 11.5	III–IV

Control	Normal Sinus Rhythm RR Interval Database	54	61.3 ± 11.8	N/A
	MIT-BIH Normal Sinus Rhythm Database	18	26–45 (5 men) 20–50 (13 women)	N/A

NYHA, New York Heart Association.

**Table 2:** Variants of PRSA for DC and AC calculation.

Method	Description
Pan et al. [15] (DC_m1_/AC_m1_)	Select anchor points on rising or falling edge of RRI signal. Equations (4) and (7) are replaced by RRI_*i*−1_ < RRI_*i*_ < RRI_*i*+1_ and RRI_*i*−1_ > RRI_*i*_ > RRI_*i*+1_, respectively.

Arsenos and Manis [16] (DC_m2_/AC_m2_)	RRI signal is represented by four successive RRI vectors. DC or AC is characterized by vector average: $\text{DC}\left(\text{AC}\right)=\left(\left({\bar{\text{RRI}}}_{i+3}+{\bar{\text{RRI}}}_{i+2}-{\bar{\text{RRI}}}_{i+1}-{\bar{\text{RRI}}}_{i}\right)/4\right)$. The vectors should satisfy ((RRI_*i*+3_+RRI_*i*+2_ − RRI_*i*+1_ − RRI_*i*_)/4) > 0 for DC and ((RRI_*i*+3_+RRI_*i*+2_ − RRI_*i*+1_ − RRI_*i*_)/4) < 0 for AC.

Nasario et al. [17] (DC_m3_/AC_m3_)	Removing points in RRI signal that change more than 20% on selecting anchor points. Equations (4) and (7) are replaced by (1 < RRI_*i*_/RRI_*i*−1_) < 1.2 and (0.8 < RRI_*i*_/RRI_*i*−1_) < 1, respectively.

**Table 3:** Optimal scale per resampling frequency and pseudofrequency corresponding to each combination of resampling frequency and scale.

Resampling frequency (Hz)	Optimal scale		Pseudofrequency (Hz)		Optimal AUC	
	DC	AC	DC	AC	DC	AC
/	2^a^	2^a^	/	/	0.920	0.818
/	4^b^	1^b^	/	/	0.940	0.917
2	6	14	0.33	0.14	0.942	0.923
3	9	21	0.33	0.14	0.939	0.923
4	13	28	0.31	0.14	0.938	0.921
5	16	34	0.31	0.15	0.936	0.921
6	20	42	0.30	0.14	0.936	0.921
7	22	49	0.32	0.14	0.936	0.921

^a^The scale was not optimized but used for conventional DC and AC calculation. ^b^The scale was optimized for DC and AC calculated from without preprocessed RRI.

**Table 4:** DC_conv_ and AC_conv_ and indices computed from preprocessed RRI signals for healthy and CHF subjects.

	CHF subjects	Healthy subjects	*p* Value
DC_conv_ (ms)	2.11 ± 2.96	6.82 ± 2.01	<0.001
DC_p_ (ms)	1.79 ± 2.16	7.37 ± 3.03	<0.001
AC_conv_ (ms)	−5.35 ± 3.60	−8.00 ± 2.56	<0.001
AC_p_ (ms)	−2.34 ± 2.10	−6.08 ± 2.17	<0.001

The preprocessing resampling frequency was 2 Hz for both DC_p_ and AC_p_, and the optimal scales were 6 and 14, respectively.

**Table 5:** Diagnostic ability of DC and AC.

	Cutoff value (ms)	Sensitivity (%)	Specificity (%)	Accuracy (%)	AUC	Ref.
DC_conv_	4.39	83.33	90.14	87.61	0.920	[10]
DC_p_	3.41	85.71	95.77	92.04	0.942	
AC_conv_	−5.81	71.43	83.10	78.76	0.818	
AC_p_	−3.33	88.10	92.96	91.15	0.923	

DC_m1_	8.24	83.33	94.37	90.27	0.947	[15]
DC_pm1_	4.50	83.33	97.18	92.04	0.940	
AC_m1_	−8.59	71.43	90.14	83.19	0.855	
AC_pm1_	−4.79	85.71	91.55	89.38	0.920	

DC_m2_	1.69	69.05	87.32	80.53	0.855	[16]
DC_pm2_	1.97	76.19	97.18	89.38	0.905	
AC_m2_	−2.20	88.10	85.92	86.73	0.894	
AC_pm2_	−2.19	83.33	91.55	88.50	0.918	

DC_m3_	4.34	78.57	94.37	88.50	0.920	[17]
DC_pm3_	3.69	78.57	92.96	87.61	0.926	
AC_m3_	−5.74	69.05	85.92	79.65	0.823	
AC_pm3_	−3.39	85.71	94.37	91.15	0.924	

Sensitivity, specificity, and accuracy of DC and AC are given under appropriate cutoff values. Subscripts m1 to m3 indicate the three calculation variants of DC and AC. The indices calculated from preprocessed RRI signals use the same resampling frequency of 2 Hz and optimal scales of 6 and 14 for DC and AC, respectively.

## Data Availability

The RR interval data used to support the findings of this study can be downloaded from http://www.physionet.org.
